# Comparative Microbiome Analysis Reveals the Ecological Relationships Between Rumen Methanogens, Acetogens, and Their Hosts

**DOI:** 10.3389/fmicb.2020.01311

**Published:** 2020-06-30

**Authors:** Zhipeng Li, Xiaoxu Wang, Antton Alberdi, Jiabo Deng, Zhenyu Zhong, Huazhe Si, Chengli Zheng, Hanlin Zhou, Jianming Wang, Yifeng Yang, André-Denis G. Wright, Shengyong Mao, Zhigang Zhang, Leluo Guan, Guangyu Li

**Affiliations:** ^1^Department of Special Economic Animal Nutrition and Feed Science, Institute of Special Animal and Plant Sciences, Chinese Academy of Agricultural Sciences, Changchun, China; ^2^College of Animal Science and Technology, Nanjing Agricultural University, Nanjing, China; ^3^Center for Evolutionary Hologenomics, GLOBE Institute, University of Copenhagen, Copenhagen, Denmark; ^4^Chengdu Zoological Garden, Chengdu, China; ^5^Beijing Milu Ecological Research Center, Beijing, China; ^6^Sichuan Institute of Musk Deer Breeding, Chengdu, China; ^7^Tropical Crops Genetic Resources Institute, Chinese Academy of Tropical Agricultural Science, Danzhou, China; ^8^College of Agricultural, Human, and Natural Resource Sciences, Washington State University, Pullman, WA, United States; ^9^State Key Laboratory of Genetic Resources and Evolution, Kunming Institute of Zoology, Chinese Academy of Sciences, Kunming, China; ^10^State Key Laboratory for Conservation and Utilization of Bio-Resources in Yunnan, Yunnan University, Kunming, China; ^11^Department of Agricultural, Food and Nutritional Science, University of Alberta, Edmonton, AB, Canada

**Keywords:** ruminants, methane, phylosymbiosis, rumen epithelium, host–microbiome interaction, reindeer

## Abstract

Ruminant methane, which is generated by methanogens through the consumption of hydrogen and supports the normal function of the rumen ecosystem, is a major source of greenhouse gases. Reductive acetogenesis by acetogens is a possible alternative sink that can dispose of hydrogen for acetate production. However, the distribution of rumen methanogens and acetogens along with the relationships among methanogens, acetogens, and their host are poorly understood. Therefore, we investigated the rumen methanogen and acetogen communities of 97 individual animals representing 14 ruminant species within three ruminant families Cervidae (deer), Bovidae (bovid), and Moschidae (musk deer). The results showed that the *Methanobrevibacter* spp. and acetogens associated with Eubacteriaceae were the most widespread methanogens and acetogens, respectively. However, other methanogens and acetogens exhibited host specificity in the rumen of reindeer and Chinese muntjac deer. Acetogen and methanogen communities were not correlated in these species, and the phylosymbiosis signature between host phylogeny and the composition of both communities was lacking. The abundance of *Methanobrevibacter gottschalkii* was negatively correlated with the degree of papillation of the rumen wall. Finally, co-occurrence analysis showed that the variation of the predicted methane yields was characterized by the interactive patterns between methanogens, acetogens, and concentrations of rumen metabolites. Our results show that rumen methanogen and acetogen communities have low compositional interdependence and do not exhibit parallel host evolution, which suggests that the strategies for mitigating methane production should be based on a species-specific rumen microbiota analysis.

## Introduction

Climate change resulting from the anthropogenic emission of greenhouse gases (GHGs), such as carbon dioxide (CO_2_), and methane (CH_4_), presents one of the major challenges the planet is facing (Jose et al., 2016). Livestock production contributes a significant amount of GHGs because ruminant species produce large amounts of CH_4_ during the digestion of plant compounds in the rumen (Gill et al., 2010; Opio et al., 2013). Thus, mitigating enteric CH_4_ emissions from ruminants urgently needs to be addressed because of the global increase in livestock production associated with human needs. Enteric CH_4_ from ruminants is mainly generated by hydrogenotrophic methanogenic archaea (i.e., methanogens) through the reduction of CO_2_ by hydrogen (H_2_). The continuous removal of H_2_ is crucial to maintaining the normal fermentative function of the rumen because excessive H_2_ accumulation inhibits carbohydrate fermentation through preventing the regeneration of NAD^+^ (Hungate, 1967; Wolin et al., 1997). Dietary additives such as secondary plant metabolites and chemical inhibitors have been shown to reduce rumen CH_4_ emissions by affecting H_2_ disposal and increasing the ratio of propionate and acetate (Kumar et al., 2014). However, these approaches may adversely influence feed intake and fiber degradability, while methanogens can also adapt to novel dietary conditions (Hristov et al., 2013). Therefore, other approaches need to be considered and developed to regulate CH_4_ emissions.

Acetogenic bacteria (acetogens) are another community of microorganisms that also remove H_2_ from the rumen ecosystem to produce energetically profitable acetate (Malik et al., 2015). The acetogens isolated from the fore-stomach of a kangaroo species (*Macropus giganteus*) with low methane emissions suggested that acetogenesis may play a major role in H_2_ removal (Ouwerkerk et al., 2009; Godwin et al., 2014). Although methanogens are thermodynamically favored to capture H_2_ when compared with acetogens (Fonty et al., 2007), recent studies have documented that acetogens largely contribute to H_2_ capture when reducing or removing methanogens. For instance, reductive acetogenesis accounted for up to 26% of H_2_ capture in methanogen-free rumen (Gagen et al., 2012), and also contributed to 21–25% to rumen fermentation in methanogen-free meroxenic lambs, which accounted for ∼66% of total H_2_ recovery (Fonty et al., 2007). Additionally, an enhancement of acetogenesis was observed during *in vitro* incubation when methanogenesis was inhibited by 2-bromoethanesulfonic acid and/or by the addition of an axenic culture of the rumen acetogen *Acetitomaculum ruminis* (le Van et al., 1998). These results suggest that reductive acetogenesis may be an alternative H_2_ sink that can reduce CH_4_ production from rumen fermentation.

Henderson et al. (2015) reported on the microbial community composition of rumen, including methanogens, across bovine species. Interestingly, deer are among the ruminants that produce relatively small amounts of CH_4_ per ingested food unit when compared with that of cattle (Pérez-Barbería, 2017). Interestingly, within the Cervidae, reindeer (*Rangifer tarandus*) is the only species distributed across the Northern Hemisphere that includes a large proportion of lichens in their diet during winter (Svein et al., 1999); this causes lower CH_4_ emissions due to the presence of secondary plant metabolites from lichens (Hansen, 2012). These results suggest that unique methanogen and acetogen communities may be present in the rumen of deer. However, the composition of rumen methanogens and acetogens of most cervids remains largely unknown. Nevertheless, increasing evidence shows that host genetic and environmental factors can affect the composition of host-associated microbiota, such as methanogens (Brooks et al., 2016; Roehe et al., 2016; Difford et al., 2018; Amato et al., 2019). A strong host genetic effect is often translated into phylosymbiosis (Brooks et al., 2016), which implies that the host-associated microbial communities are parallel to the phylogeny of the host species (Brooks et al., 2016). Using such an approach, co-evolution has been observed within the microbiota of the mammalian gut (Moeller et al., 2016), and skin microbiota (Ross et al., 2018). However, no study has yet examined the phylosymbiosis of rumen methanogens and acetogens. Therefore, unveiling the composition and interactions between rumen methanogens and acetogens, and the evolutionary histories between different ruminant families and their rumen microbiomes, might contribute to designing manipulation strategies for decreasing CH_4_ emissions through the targeting of the key methanogen(s) in the rumen.

In this study, we analyzed the rumen methanogen and acetogen communities of 11 deer species (Cervidae) and compared them to two bovids (cattle and sheep) and one moschid (forest musk deer). We first characterized the taxonomic composition of methanogenic and acetogenic species in rumen to test whether communities differed between species and identified the common and host-specific features. Second, we examined whether rumen methanogen and acetogen communities displayed parallel arrangements and if the compositional variations observed among hosts mirror host phylogenies. Finally, we used co-occurrence networks to elucidate whether the relationship between methanogens and acetogens changed according to the predicted CH_4_ emission of hosts.

## Materials and Methods

### Animals and Sampling

All animal-specific procedures were approved and authorized by the Chinese Academy of Agricultural Sciences Animal Care and Use Committee, and the Institute of Special Animal and Plant Sciences Wild Animal and Plant Subcommittee (No. ISAPSAEC-2019001L). A total of 97 animals from 14 ruminant species belonging to the families Cervidae, Moschidae, and Bovidae were used in the present study (Supplementary Table S1). The animals included white lipped deer (*Cervus albirostris*, *n* = 8), sika deer (*Cervus nippon*, *n* = 5), red deer (*Cervus elaphus*, *n* = 5), fallow deer (*Dama*, *n* = 8), Chinese water deer (*Hydropotes inermis*, *n* = 8), Chinese muntjac deer (*Muntiacus reevesi*, *n* = 8), milu deer (*Elaphurus davidianus*, *n* = 6), Eld’s deer (*Cervus eldii*, *n* = 5), sambar (*Rusa unicolor*, *n* = 8), hog deer (*Axis porcinus*, *n* = 6), reindeer (*R. tarandus*, *n* = 8), forest musk deer (*Moschus berezovskii*, *n* = 7), Tibetan sheep (*Ovis aries*, *n* = 8), and cattle (*Bos taurus*, *n* = 7). Among these species, the white lipped deer, sika deer, red deer, Chinese muntjac deer, milu deer, Eld’s deer, reindeer, and Tibetan sheep grazed in local pastures or on leaves (Supplementary Table S1). Because the animals within the Cervidae and Moschidae families were vigilant to human activity and have a strong wild nature, the animals were first anesthetized; then, rumen liquids were collected *via* stomach tubing before morning feeding (captive species) or grazing (wild species). In order to avoid saliva contamination, the first 100 ml of rumen fluid was discarded, and then, approximately 50 ml of rumen liquid was collected. The rumen samples were immediately instantly frozen using liquid nitrogen and transferred to the laboratory where they were stored at −80°C until performing the analysis.

### DNA Extraction, Amplification, and High-Throughput Sequencing and Analysis

Total genomic DNA was extracted from each sample using a QIAamp DNA Stool Mini Kit (Qiagen, Chatsworth, CA, United States) with some modifications. Briefly, ∼200 mg of rumen liquid was added to a 2.0-ml tube containing glass beads of 1. 4-, 0. 1-, and 4-mm diameter (Lysing Matrix E, MP Biomedicals, Illkrich, France), and then 1.4 ml of ASL buffer from a QIAamp DNA Stool Mini Kit (Qiagen) was added. Samples were immediately subjected to bead beating for 45 s at a speed of 6.5 m/s using a FastPrep-24 (MP Biomedicals, Illkrich, France). Next, each tube containing a sample and ASL buffer was incubated at 95°C for 7 min for lysis. Subsequent steps of the DNA extraction followed the manufacturer’s instructions (Qiagen).

Primers Met 86F (Wright and Pimm, 2003) and 519R (Turner et al., 1999) were used to amplify the methanogen 16S rRNA genes (*ca.* 380 bp), and primers ACS_f (5′-CTBTGYGGDGCIGTIWSMTGG-3′) and ACS_r (5′-AARCAWCCRCADGADGTCATIGG-3′) were used to amplify the acetogens’ specific subunit B of the acetyl-CoA synthase gene (*acsB*, *ca.* 216 bp; Gagen et al., 2010). The forward primer contained an addition of a six-base barcode, and each primer contained an Illumina adapter sequence. The resulting amplicons were purified using a QIAquick PCR Purification Kit (Qiagen) and subjected to library construction. A PhiX Control library (Illumina, 20%) was combined with the amplicon library, and samples were then sequenced on an Illumina PE MiSeq platform to generate paired 300-bp reads. Sequence Read Archive accession numbers for the sequences reported in this paper are SRP097265 and SRP097328.

Sequences were processed using QIIME 1.9.0 (Caporaso et al., 2010). For methanogens, quality-filtered sequences were clustered into operational taxonomic units (OTUs) using Usearch61 according to a sequence similarity of 97%. For acetogens, the *acsB* nucleotide sequences were translated into amino acid sequences based on the approach of Yang et al. (2016) using a validated reading frame and ExPASy (Gasteiger et al., 2003). The *acsB* amino acid sequences were clustered into OTUs at a distance of ≤0.035 (96.5% identity) using Cluster Database at High Identify with Tolerance software (Li and Godzik, 2006).

### Real-Time PCR Quantification for Rumen Bacteria, Methanogens, Acetogens, and Protozoa

Specific primers (Supplementary Table S2) targeting for the methyl coenzyme-M reductase A gene (*mcrA*; Denman et al., 2007) and the formyltetrahydrofolate synthetase gene (*fhs*; Xu et al., 2009) were applied to amplify methanogens and potential acetogens, respectively. The 16S rRNA gene of the total bacteria and the 18S rRNA gene of total protozoa were also amplified using the primers (Supplementary Table S2) as reported in previous studies (Sylvester et al., 2004; Kwong et al., 2017). Real-time PCR was performed with SYBR green chemistry using ABI7500 (Life Technologies, Singapore). The PCR program used involved one cycle of initial denaturation at 95°C for 10 s, followed by 40 cycles of denaturation at 95°C for 15 s and annealing at 60°C for 1 min. The PCR amplicons were cloned using αpGEMR T Easy kit (Promega, Shanghai, China) following the method reported by Koike et al. (2007) to obtain the plasmid DNA containing the respective standard. The standards were prepared from serial dilutions of the linearized plasmid containing between 10^2^ and 10^9^ target genes (bacterial and methanogens 16S rRNA genes, acetogens *fhs* genes, and protozoa 18S rRNA genes) with copies calculated from the concentration of plasmids. The copy numbers of the standards and the target genes were calculated according to the method used by Ritalahti et al. (2006).

### Measuring the Volatile Fatty Acids (VFAs) in the Rumen of 14 Ruminant Species

Rumen fluid was centrifuged at 15,000 × *g* for 10 min at 4°C, and then 0.2 ml of HPO_3_ (25% w/v) was added to 1 ml of clarified rumen fluid for VFA measurement based on gas chromatography using a flame ionization detector and a DB-FFAP column (30 m × 0.25 μm × 0.25 μm; 6890GC; Agilent Technologies, Inc., Santa Clara, CA, United States). The carrier gas was N_2_ at a flow rate of 2.2 ml/min. The analysis was a gradient oven temperature of 80–170°C with an incremental rate of 10°C/min for optimal separation and a detector temperature of 250°C. The estimated CH_4_ production in rumen contents of animals was calculated from the relationship between CH_4_ and VFAs as described previously (Gagen et al., 2012): methane = 0.45 acetate-0.25 propionate + 0.40 butyrate.

### Host Phylogeny and Microorganism Compositional Congruency Analyses

Principal coordinates analysis (PCoA) was used to compare the methanogen and acetogen communities in the rumen across all hosts using R software (version 3.5.0). Bayesian phylogenetic trees were constructed from the complete mitochondrial sequences of the 14 ruminant species downloaded from GenBank using Beast 2.4 (Drummond et al., 2012) and were generated in two independent Markov chains with an uncorrelated log-normal relaxed molecular clock, a Yule speciation prior, and 5 million generations sampled every 1,000 states. The Markov chains were visualized in Tracer 1.5^1^ to ensure that both independent chains had converged, and the estimated sample size values of all parameters were above 500. The composition dendrograms for microbiota were generated using Bray–Curtis and weighted and unweighted UniFrac distances by a custom iterative pipeline that incorporated the phylogenetic uncertainty of microorganisms.

A phylosymbiosis index (PI) was calculated for each community based on a previous study (Brooks et al., 2016), by averaging the topology comparisons of the host phylogenetic tree with the composition dendrograms of 1,000 methanogens/acetogens. The index was computed using each of the 1,000 phylogenetic trees as the complement of the division between the Robinson–Foulds (RF) topological distance between the trees (RF_i_) and the maximum possible RF distance (RF_max_) for a tree with 14 taxa [1-(RF_i_/RF_max_)]. Thus, the PI ranges from 0 to 1, where 0 indicates no phylogenetic signal in the microbial community composition (completely different topology) and 1 indicates perfect phylosymbiosis (identical topology). For visualization, host phylogeny and microbial composition tree topologies were compared using the *tanglegram* function in the R package *dendextend* (Galili, 2015).

### Interpretive Analysis Between Rumen Wall Structure and Microbial Composition

A surface enlargement factor (SEF) was applied to reflect the degree of basal mucosal surface gain caused by papillation of the rumen wall (Schnorr and Vollmerhaus, 1967). The SEF data of the atrium as well as the dorsal and ventral surfaces of the rumen were obtained from the study by Clauss et al. (2009), except for red, white-lipped, and forest musk deer due to a lack of data. The species distance matrices generated from microbial composition and SEF data were compared using mantel and Pearson’s product moment correlation tests. The correlation between the relative abundances of the dominant methanogens or acetogens and the mean SEF values of the dorsal and ventral surfaces was also calculated (Supplementary Materials and Methods).

### Microbial Community and Metabolite Relationship

Redundancy analysis (RDA) was performed to identify the relationship between rumen methanogens, acetogens, and metabolites. Co-occurrence network analysis was used to examine the existence of correlations among the methanogens, acetogens, and metabolites across all hosts (Li et al., 2015). Network analyses were carried out using Cytoscape 2.8.2 with a force-directed algorithm (Smoot et al., 2011). The networks were analyzed and compared using the DyNet neural network software (Goenawan et al., 2016), to uncover how the networks were physically rewired. All values are expressed as the mean unless otherwise stated (Supplementary Materials and Methods).

## Results and Discussion

Microbial manipulation designed to favor acetogens to the detriment of methanogens has been proposed as an approach to achieve the goal of decreasing CH_4_ emissions from grazing animals (Weimer, 2015; Hill et al., 2016). However, the development of effective microbial manipulation strategies that are designed to decrease CH_4_ production requires an understanding of the composition of methanogen and acetogen communities in different ruminant species, as well as an understanding of the relationship of rumen methanogens and acetogens with the host. In this study, the composition of ruminal methanogen and acetogen communities of 14 species was compared, aiming to provide an in-depth understanding of their role in predicted CH_4_ emissions.

### Methanogen and Acetogen Communities Differ Among Host Species

**FIGURE 1:**
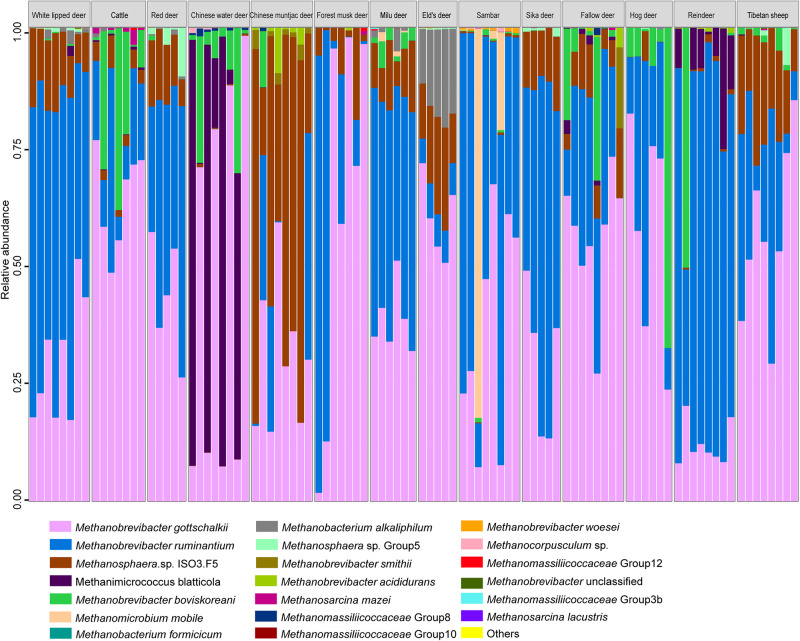
Methanogen community composition at the species level in the rumen of 14 ruminant species.

**FIGURE 2:**
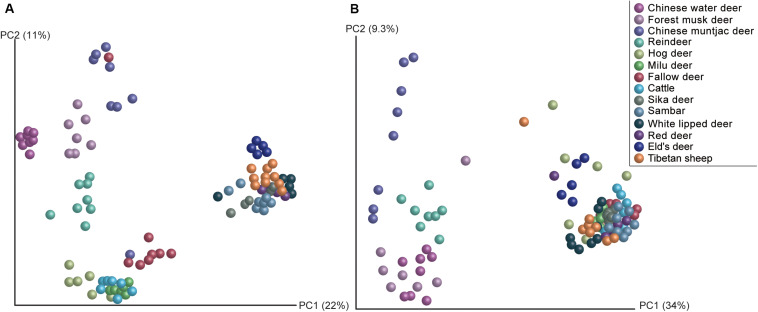
Comparisons of the methanogen and acetogen communities in the rumen of 14 species. **(A)** Principal coordinates analyses based on the Bray–Curtis distance of methanogens in the rumen of 14 ruminant species. **(B)** Principal coordinates analyses based on the Bray–Curtis distance of acetogens to reveal the changes of community structure with regard to ruminant species.

Methanogen communities of 97 rumen samples from 14 ruminant species were characterized by sequencing the V1–V3 regions of PCR-amplified 16S rRNA genes. Overall, a total of 887 OTUs representing 20 methanogen species were obtained from the 845,854 quality-filtered sequences (Figure 1). Methanogen communities were generally dominated by two species (Simpson’s dominance index: 0.52 ± 0.15): *Methanobrevibacter gottschalkii* and *Methanobrevibacter ruminantium*, accounting for more than 79% of the methanogen OTUs detected in the rumen of all analyzed hosts, although a great amount of variation was observed in each ruminant species. Similar to the findings of bovines, caprids, camelids, and *Cervus elaphus* (Henderson et al., 2015), these two methanogenic species also dominate the rumen methanogen communities of most cervid species, suggesting the core and predominant role of the two species in rumen ecology. However, the composition of the methanogen community differed among analyzed host species (Figure 2A, PERMANOVA: *F* = 20.544, *df* = 13, and *p* < 0.001). In fact, the Chinese water deer, Chinese muntjac deer, and reindeer exhibited the most diverse methanogen compositions when compared with all other species analyzed here (Figures 1, 2A). The relative abundance of *Methanomicrococcus blatticola* was relatively higher in the rumen of Chinese water deer (43.1%) and reindeer (8.6%), which is far above the marginal values (0.02–0.69%) previously reported for other ruminants (Wright et al., 2004; Huang et al., 2016). In addition, the rumen methanogen community of Chinese muntjac deer was also dominated by *Methanosphaera* sp. ISO3-F5 (50.9%). *Methanomicrococcus blatticola* is an H_2_-dependent methylotroph methanogen that requires lower H_2_ levels to produce CH_4_ and has the ability to remove oxygen and thus establish appropriate anaerobic conditions for its own survival and growth (Sprenger et al., 2000, 2007). Similarly, this methanogen was detected in epithelial samples of cattle but was virtually undetected in rumen bulk samples (De Mulder et al., 2017). Moreover, *Methanomicrococcus blatticola* also showed a relatively high efficiency of energy conservation as reflected by the growth yields (Sprenger et al., 2007). These results suggest that *Methanomicrococcus blatticola* plays a role in oxygen removal and is likely to be associated with energy metabolism in the rumen ecosystem of Chinese water deer and reindeer. *Methanosphaera* spp. also can convert ethanol to acetate through putative alcohol and aldehyde dehydrogenases when the amount of free H_2_ is small, which would produce the required protons and electrons to reduce methanol groups to methane (Hoedt et al., 2016). The genes responsible for the alcohol and aldehyde dehydrogenases in the rumen of Chinese muntjac deer warrant further examination using a metagenome approach. Chinese water and muntjac deer are the two species that exhibit the most intense browsing habits, while the other studied species are mainly grazers. These two species commonly consume herbaceous and woody material, including tannin-enriched leaves, resulting in the production of some short-chain alcohols during fermentation (Cantalapiedra et al., 2014). We also previously observed that the proportion of *Methanosphaera* spp. increased when sika deer was fed on tannin-rich plants (Li et al., 2013). Thus, these results suggest that the dietary habits of the host could be an important factor in determining the community structure of rumen methanogens. However, the effect of dietary components on rumen microbiota, which provides the substrates to methanogens (Kamke et al., 2016), is also important, and these should be further examined and compared.

**FIGURE 3:**
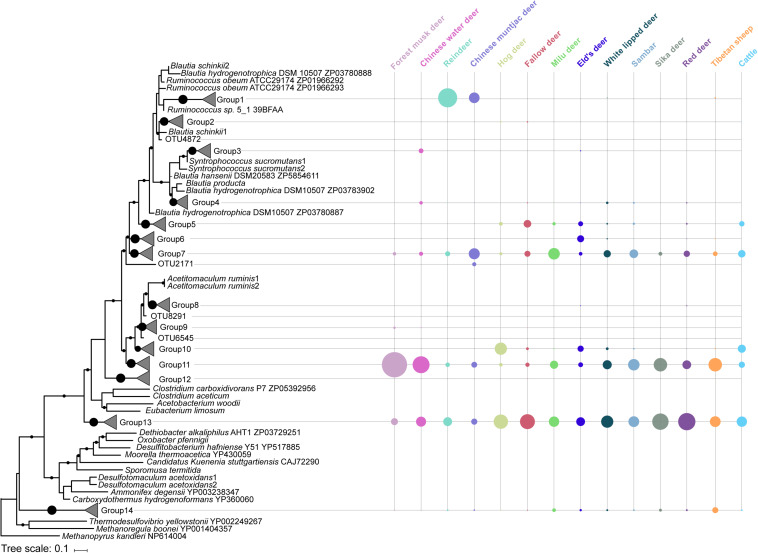
Acetogen composition based on the phylogenetic analysis of acetyl-CoA synthase amino acid sequences in the rumen of 14 ruminant species. Bootstrap values greater than 75% are shown at the nodes. The scale bar represents a sequence divergence of 10%. The node size on the right side represents the relative abundance of each group or operational taxonomic unit (OTU).

Rumen acetogen community was examined by sequencing and analyzing the *acsB* gene. A total of 192 OTUs were from the 1,530,546 high-quality amino acid sequences that corresponded to the 14 groups based on phylogenetic affiliation (Figure 3). Acetogen communities were generally comprised of two dominant groups (Simpson’s dominance index: 0.27 ± 0.10), Groups 13 and 11, belonging to families Eubacteriaceae and Clostridiaceae, respectively (Figure 3). These two bacterial families have been widely identified as occurring in the gastrointestinal tracts of herbivorous animals. For example, *Eubacterium limosum*, within the family Eubacteriaceae, was the first acetogen isolated from sheep rumen (Genthner et al., 1981). *Clostridium* spp. has been detected in the rumen of most ruminants (Gagen et al., 2012; Li et al., 2017) and in the cecum of other herbivorous mammals such as rabbits (Yang et al., 2016). These results suggest that greater diversity may exist among acetogens in the rumen. However, it is possible that the reductive acetogens identified in our study are missing the complete reductive acetogenesis pathway. Thus, it is worth constructing the metagenome assembly genomes of the reductive acetogens identified in this study and examining their complete reductive acetogenesis pathway.

The composition of acetogens also differed among the analyzed ruminant species (Figure 2B, PERMANOVA: *F* = 8.502, *df* = 13, and *p* < 0.001), and inter-specific variability explained 57.1% of the total compositional variation. Reindeer exhibited a different composition dominated by Group 1, placed phylogenetically between the *Blautia* group and the Ruminococcaceae (Figure 3). *Blautia* spp. play an important role in fermentation in the kangaroo foregut, can convert CO_2_ to acetate, and produce propionate through the conversion of pyruvate, oxaloacetate, malate, fumarate, and succinate (Godwin et al., 2014); *Blautia* spp. are the abundant members in the rumen of isolated lambs without rumen methanogens (Gagen et al., 2012). Gagen et al. (2015) also demonstrated that the abundance of acetogens affiliated with Ruminococcaceae decreased when the culture medium was lacking H_2_, which also enriched the genes involved in the metabolism of pyruvate and acetyl-CoA (Medvecky et al., 2018). These findings indicate that the acetogens classified in Group 1 could be active in pyruvate metabolism and acetate production; thus, reindeer could be ideal sources for isolating rumen acetogens. Obtaining and characterizing isolates from Group 1 and analyzing their genomes will be important in shedding light on their activity and function in the rumen.

### Acetogens and Methanogens Exhibit Diversity but Not Compositional Correlation

**FIGURE 4:**
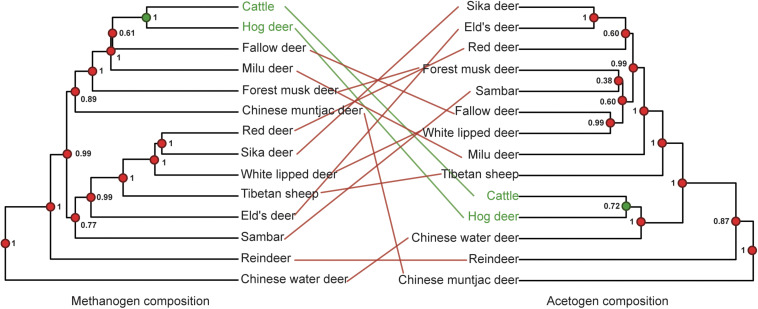
Co-occurrence analysis of methanogens and acetogens. Node dot color indicates whether the node contains the same (green) or a different (red) combination of taxa as the paired tree. Node values indicate posterior probabilities in the host phylogeny and node support based on 1,000 iterations of UniFrac distances in microbiota composition dendrograms. The color of lines connecting the taxa indicates whether taxa are located in identical (green) or different (red) subtrees in both trees.

The diversity and richness of methanogen and acetogen communities showed similar patterns (Supplementary Figure S1). Interestingly, the averaged Shannon diversity index of methanogen and acetogen communities per species was positively correlated (Pearson’s correlation test: *r* = 0.79, *t* = 4.47, *df* = 12, and *p* < 0.001; Supplementary Figure S2). In addition, the numbers of total bacteria, protozoa, methanogens, and acetogens were also not significantly different in the rumen among the analyzed ruminant species based on real-time PCR (corrected *p* > 0.05), with the exception of protozoa and methanogens in rumen of Chinese muntjac deer (Supplementary Figure S3). However, the dendrograms based on species-level microbial composition exhibited very low co-occurrence between acetogen and methanogen communities (Figure 4). This suggests the lack of directed dependencies between specific methanogen and acetogen species in the rumen, which is likely related to the different colonization and development histories of various species (Skillman et al., 2004), the higher H_2_ utilization threshold of acetogens (Cord-Ruwisch et al., 1988), micro-niches (Leadbetter et al., 1999), and population densities between methanogens and acetogens (Joblin, 1999; Wright and Klieve, 2011). Overall, these results revealed that an inconsistent co-occurrence pattern occurred in the diversity and composition between methanogen and acetogen communities in the rumen.

### Acetogen and Methanogen Communities do Not Exhibit Host Phylogenetic Signal

To test the relationship between host phylogeny with rumen methanogens and acetogens, we conducted a phylosymbiosis analysis, which would lead to closely related species being associated with similar microbial communities (Brooks et al., 2016). This effect can be measured using the RF value, which compares the congruence between two phylogenetic trees.

**FIGURE 5:**
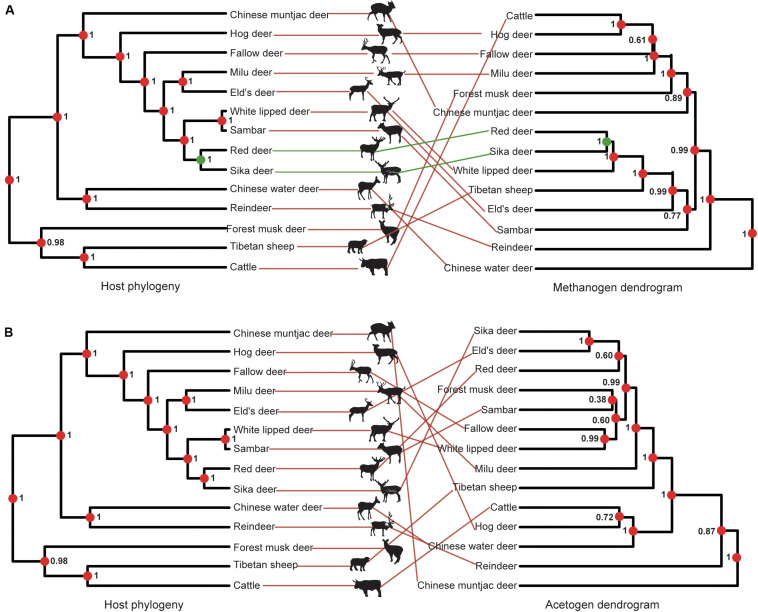
Phylosymbiosis between host phylogeny and microbiota composition dendrograms. **(A)** Phylosymbiosis between host phylogeny and methanogen composition dendrograms. **(B)** Phylosymbiosis between host phylogeny and acetogen composition dendrograms. Node dot color indicates whether the node contains the same (green) or a different (red) combination of taxa as the paired tree. Node values indicate posterior probabilities in the host phylogeny and node support based on 1,000 iterations of UniFrac distances in microbiota composition dendrograms. The color of lines connecting the taxa indicates whether taxa are located in identical (green) or different (red) subtrees in both trees.

Methanogens barely showed any congruence with a PI score of 0.19 ± 0.002 (unweighted unifrac distance) because the closely related red and sika deer were the only species properly paired (Figure 5A). The PI scores based on weighted unifrac and Bray–Curtis distances were 0.01 ± 0.01 and 0.00 ± 0.00, respectively. The incongruence between acetogen composition and host phylogeny was even more remarkable, with a PI of 0.01 ± 0.01 (Figure 5B). The PI scores based on weighted unifrac and Bray–Curtis distances were both 0.00 ± 0.00. Rumen methanogen and acetogen communities do not consequently parallel the phylogeny of host species, contrasting with previously reported observations of gut bacteria based on fecal samples of 24 animal species from four different groups (*Peromyscus* deer mice, *Drosophila* flies, mosquitoes, and *Nasonia* wasps) that span a range of estimated divergence times from 0.2 to 108 million years (Brooks et al., 2016) and 33 mammals with an evolutionary time up to 80 million years (Groussin et al., 2017). A previous study demonstrated that the magnitude of phylosymbiosis decreased with increasing evolutionary time (Groussin et al., 2017). Our study also revealed the Bovidae and Cervidae diverged about 20 million years ago (Chen et al., 2019), which may partially explain the unobserved signal of phylosymbiosis in our study. Moreover, this incongruence might also be caused by the specific features of the different communities analyzed between the mentioned studies (bacteria) and the present study (methanogen and acetogen), as well as samples (gut feces vs. rumen liquid). In addition, the fecal samples represent the microbial composition of the large intestine (i.e., colon). As opposed to the colon, the rumen is located in the beginning of the intestinal tract, which is more exposed to environmental factors (Dominguez-Bello et al., 2010; Zhou et al., 2014). Hence, this observation supports the idea that rumen methanogen composition is largely conditioned by environmental factors (Henderson et al., 2015). Additionally, rumen methanogens have been shown to develop symbiotic relationships with bacteria, protozoa, and fungi in specialized anaerobic rumen environment (Janssen and Kirs, 2008). Thus, rumen methanogens may be affected by environmental transmission resulting in the limited congruence of the phylogeny. It is possible that congruence would be observed if the ruminants were sampled at similar time points in their lifespan and inhabited the same geographic location. No significant phylosymbiosis was observed for acetogens, suggesting that the functional gene (*acsB*) using the acetyl-CoA pathway for producing the acetate as a reduced product of CO_2_ and H_2_ is not determined by host phylogeny. Because CH_4_ production is a heritable trait with a ruminant host (Pinares-Patiño et al., 2013; Lassen and Løvendahl, 2016), these findings reveal that methanogen or acetogen community composition may not be good markers in predicting CH_4_ emissions. However, the low phylosymbiosis of methanogens associated with hosts indicates the limited role of host genetic factors in affecting the composition of methanogens; it is possible to mitigate CH_4_ emissions through dietary or microbial manipulation approaches because the methanogens are the only producer of methane in the rumen.

### Acetogen Communities and Core Methanogen Abundance Correlate With Rumen Wall Morphology

A previous study has demonstrated that host physiology is an important factor in shaping the gut microbiota (Amato et al., 2019), and gut fermentation physiology also significantly affected microbial diversity (McKenzie et al., 2017); thus, we further explored the relationship between methanogen and acetogen communities with hosts. We examined whether the observed inter-specific community variations are related to rumen morphological differences across species by comparing the species-specific rumen wall SEF, which indicate the degree of papillation pattern of the rumen wall. Briefly, for grazing ruminants, such as cattle and sheep, the rumen contents are stratified, consisting of a distinct gas fraction including CO_2_ and CH_4_ above the fibrous materials, which, itself, floats on the liquid layer. In contrast, for browsing ruminants, such as Chinese water deer and moose, the rumen contents appear to be rather homogenous, without an obvious separation of gas, particles, and fluid (Clauss et al., 2009). The generated VFAs can be assumed to be relatively evenly distributed throughout rumen contents, resulting in an even ruminal papillation. Thus, the production of VFAs promotes rumen papillation, while the presence of a dorsal gas dome could prevent papillae formation in these locations; this occurs because gas and sludge will displace any VFAs that could accumulate in these regions. The SEF value represents the factor by which the absorptive surface of the rumen mucosa is increased due to the papillae in comparison to the basal rumen area; an unpapillated region of the rumen, thus, has an SEF of 1.0 (Clauss et al., 2009).

The acetogen community structure at the OTU level exhibited a significant correlation with SEF (mantel test: *r* = 0.423, *p* = 0.013; Pearson’s correlation test: *r* = 0.42, *t* = 3.340, *df* = 53, and *p* = 0.001), suggesting that a possible correlation exists between the rumen epithelium morphology and the acetogen community membership. The real mechanism is worth further examination, such as an analysis of the relationship between acetogen composition and epithelial microbiota. In contrast, the relative abundance of the dominant methanogen, *M. gottschalkii*, exhibited a negative correlation with SEF (Pearson’s correlation test: *r* = −0.77, *t* = −3.66, *df* = 9, and *p* = 0.005). Grazing ruminants usually have rumen with lower SEF values and release higher amounts of H_2_ due to the increased amount of fiber digestion compared to browsing ruminants that have higher SEF scores (Pérez-Barberia et al., 2004). The genome analysis of *Methanobrevibacter millerae* SM9 belonging to the *M. gottschalkii* clade showed that this methanogen has a larger complement of genes involved in methanogenesis including genes for methyl coenzyme M reductase II (*mrt*; Kelly et al., 2016). The relative abundance of *M. gottschalkii* and the expression of *mrt* increased in the high CH_4_ yield sheep compared with the low CH_4_ yield sheep (Shi et al., 2014). This may explain the negative correlation between *M. gottschalkii* and SEF.

### Metabolite–Microorganism Interactive Relations Under Different Host Environments

**FIGURE 6:**
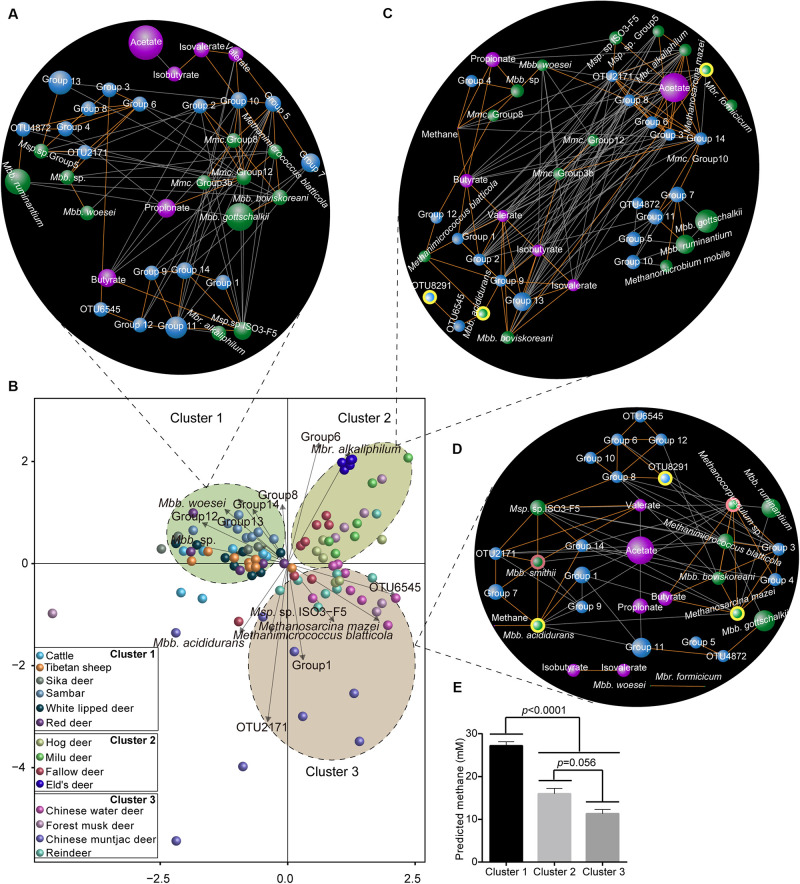
Interactive relationships between methanogens, acetogens, and rumen metabolites. **(A)** Redundancy analysis showing the significant microorganisms (*p* < 0.05) driving the rumen fermentation products across all the hosts based on the permutation test. Co-occurrence network of the rumen methanogens, acetogens, and fermentation parameters in cluster 1 (**B**, high CH4 yield group), cluster 2 (**C**, moderate CH4 yield group), and cluster 3 (**D**, low CH4 yield group). Blue, green, and purple nodes represent acetogens, methanogens, and metabolites, respectively. Node size indicates the relative abundance of microorganisms or percentage of metabolites. The nodes with light yellow border represents the commonly changed microorganisms in networks 2 and 3 compared to network 1, and the nodes with a pink border indicate the changed microorganisms in network 3 compared to network 2. Each co-occurring pair has an absolute Spearman rank correlation above 0.50 [Gold line: positive correlation (*r* > 0.90); gray line: negative correlation (*r* < -0.50)] with an FDR-corrected significance level under 0.01. **(E)** Bar plots showing the amount of predicted CH_4_ yields in three clusters. *Mbb*, *Methanobrevibacter*; *Msp*, *Methanosphaera*; *Mbr*, *Methanobacterium*; and *Mmc*, *Methanomassiliicoccaceae*.

We first used RDA to interpret the relationship between microorganism and metabolites (Supplementary Tables S3, S4), and found that species were clustered into three clusters (*p* < 0.05; Figure 6A). Through co-occurrence network analysis, we analyzed the different metabolite–microorganism relationships in light of predicted CH_4_ yields. First, in cluster 1 (the high CH_4_ yield species group), acetate was not associated with any of the identified microorganisms (Figure 6B). In contrast, in cluster 2 (the moderate CH_4_ yield group), acetate was positively correlated (*r* = 0.57–0.69, *p* = 8.1 × 10^–6^ − 0.0004) with diverse acetogens (e.g., *Blautia* group, Lachnospiraceae, and Clostridiaceae; Figure 6C), while in cluster 3 (the low CH_4_ yield group), acetate was negatively correlated with acetogen OTU 2171 (*r* = -0.56; *p* = 0.001), propionate (*r* = -0.56; *p* = 0.0009), and valerate (*r* = -0.58; *p* = 0.0006; Figure 6D). These findings indicate the relationship between the molar percentage of acetate and acetogens varies with the predicted CH_4_ production. These relationships are difficult to interpret, however, because rumen acetate is produced from the metabolism of carbohydrates, and the magnitude of measured acetate produced by reductive acetogenesis is unknown.

Second, the interactive pattern between methanogens and predicted CH_4_ also varied among the three clusters. In cluster 1, the predicted CH_4_ yield was not associated with any identified microorganisms (Figure 6B). In contrast, the predicted CH_4_ amount was negatively correlated with *Methanobacterium alkaliphilum* (*r* = -0.51; *p* = 0.002) in species from cluster 2 (Figure 6C), while it was positively correlated with *Methanobrevibacter acididurans* (*r* = 0.53; *p* = 0.002) in species from the cluster 3 (Figure 6D). These results also indicate that the possible role of methanogens in rumen predicted CH_4_ yields and might vary based on the host species. However, the dietary effect on rumen methanogens and methane production was not excluded in our study (Kumar et al., 2014), although a rumen content transplant study on dairy cows showed that restoration of the rumen microbial composition was influenced by the host (Weimer et al., 2010; Foster et al., 2017). Together, these findings suggest that microbial manipulation strategies to decrease CH_4_ yields should consider the composition of host-specific methanogens because of the species-specific characteristics of CH_4_ metabolism (Pérez-Barbería, 2017). In contrast, interactions between some low abundance methanogens such as *Methanobrevibacter acididurans*, *Methanosarcina mazei*, and *Methanobacterium formicicum*, and predicted CH_4_ yield were only found in species with moderate (Figure 6C) and low (Figure 6D) CH_4_ production. Previous studies have shown that some methanogen species with low abundances (*Methanobrevibacter* spp., 0.1–1.8%) were evenly distributed and, thus, could also be connected to the host’s metabolism requirements (Wallace et al., 2019). Although some microbes are low in abundance, they play important roles in the complete metabolic potential and in shaping the gut microbial community when they are highly active, enhanced, trigger the metabolic activity of more dominant members, or if they contain enzymes needed for complex metabolic processes (Jousset et al., 2017; Benjamino et al., 2018). Moreover, the predicted rumen CH_4_ yield is closely related to the methanogen composition at the species and/or strain levels (Zhou et al., 2010; Danielsson et al., 2017). Therefore, these findings suggest that some methanogens with rare and/or low abundance play a role in CH_4_ emissions, thus, providing a potential target for designing microbial methods to manipulate rumen CH_4_ production. However, the CH_4_ emissions were estimated by a stoichiometric equation, which could be biased. Measuring the amount of H_2_, CO_2_, and CH_4_ produced by ruminants using respiration chambers in the future is needed to verify the roles of methanogens and acetogens in enteric methane emissions.

## Conclusion

The present study characterized the methanogen and acetogen community composition in 14 ruminant species and identified significant compositional differences among them. The diversity of acetogens and methanogens was positively correlated, but both communities exhibited no compositional interdependence across the studied ruminant species. The low host phylogenetic signal of both communities indicates that these two groups of microorganisms had different evolutionary patterns, suggesting the possible limited role of host genetic effects on rumen methanogens and acetogens. However, the composition of acetogen communities and the abundance of *M. gottschalkii* were significantly correlated with SEF scores, suggesting that the degree of papillation of the rumen epithelium might be closely related to the composition of both acetogens and abundant methanogens. Moreover, the co-occurrence between methanogens, acetogens, and predicted CH_4_ in rumen changed with the differently predicted CH_4_ yields, addressing the importance of the complicated interaction between host with methanogen and acetogen abundance as well as the community composition for CH_4_ yield. Unveiling the interactions between rumen methanogens and acetogens in the rumen of species from the low methane cluster, such as reindeer, might contribute to design manipulation strategies. Importantly, the metagenome approach should be further applied to examine the metabolic pathways of methane and acetate production.

## Data Availability Statement

The datasets generated for this study can be found in the Sequence Read Archive (SRA) accession number for the sequences reported in this article are SRP097265 and SRP097328, respectively.

## Ethics Statement

The animal study was reviewed and approved by Chinese Academy of Agricultural Sciences Animal Care and Use Committee, and the Institute of Special Animal and Plant Sciences Wild Animal and Plant Subcommittee.

## Author Contributions

ZL designed the study. ZL, XW, JD, ZhoZ, HS, and SM collected the samples. CZ, HZ, JW, and YY assisted in collecting the samples. ZL and AA analyzed the data and wrote the manuscript. A-DW, ZhaZ, LG, and GL revised the manuscript. All authors contributed to the article and approved the submitted version.

## Conflict of Interest

The authors declare that the research was conducted in the absence of any commercial or financial relationships that could be construed as a potential conflict of interest.

## Footnotes

1 http://tree.bio.ed.ac.uk/software/tracer/

## Supplementary Material

FIGURE S1 The diversity and richness indices of acetogens **(A)** and methanogens **(B)** in the rumen of 14 species. ^∗^*p* < 0.05, ^∗∗^*p* < 0.01, and ^∗∗∗^*p* < 0.001.

FIGURE S2 Correlation of the Shannon diversity index of methanogen and acetogen communities in the rumen of 14 species.

FIGURE S3 Density of the selected microbes in the rumen of 14 ruminant species including total bacteria **(A)**, protozoa **(B)**, methanogens **(C)**, and acetogens **(D)**. Abundance was expressed as log_10_ copies of target gene per gram of wet weight. Target gene: 16S rRNA gene for bacteria; *mcrA* for methanogens; 18S rRNA for protozoa; *fhs* for acetogens. The different letters (a, b) denote significant differences (Kruskal–Wallis tests, FDR-adjusted *p* < 0.05).

TABLE S1 The distribution, habit, diet, and management of the ruminant species used in the present study.

TABLE S2 Primer sets used to quantify the rumen microbiota of 14 ruminant species.

TABLE S3 Comparison of the percentage of rumen volatile fatty acids (VFAs) among the 14 ruminant species.

TABLE S4 The detailed comparison results of rumen VFAs among the 14 ruminant species.
