# Supplementary material for: Comparative Microbiome Analysis Reveals the Ecological Relationships Between Rumen Methanogens, Acetogens, and Their Hosts
Source: Front Microbiol. 2020 Jun 30;11:1311. doi: 10.3389/fmicb.2020.01311 (PMC7344211; doi:10.3389/fmicb.2020.01311)
Supplement: FIGURE S1 — The diversity and richness indices of acetogens (A) and methanogens (B) in the rumen of 14 species. ∗p < 0.05, ∗∗p < 0.01, and ∗∗∗p < 0.001. [file Data_Sheet_1.DOCX]

Supplementary Material

# Detailed materials and methods

**Sequences analysis of methanogens and acetogens**

Sequences were processed using QIIME 1.9.0 (Caporaso et al., 2010). For methanogens, quality-filtered sequences were clustered into operational taxonomic units (OTUs) using Usearch61 according to a sequence similarity of 97% at the species level for the methanogens 16S rRNA gene. Potential chimeric sequences were removed using Chimera Slayer (Haas et al., 2011). OTU representative sequences were aligned to the RIM-DB for taxonomy assignment (Seedorf, Kittelmann, Henderson, & Janssen, 2014). For acetogens, the *acsB* nucleotide sequences were translated into amino acid sequences in a specific open reading frame using ExPASy (Gasteiger et al., 2003). The *acsB* amino acid sequences were clustered into OTUs at a distance of ≤ 0.035 (96.5% identity) using the CD-HIT (W. Li & Godzik, 2006). A maximum likelihood tree was constructed to determine the phylogenetic relationship of acetogens based on *acsB* OTUs as described by Gagen et al. (Gagen et al., 2010). The sequencing depth was scaled down to 8,000 sequences by random sampling to ensure sample comparability. OTUs present in at least 50% of the samples representing the core OTUs were retained to further analysis. Alpha-diversity of each sample including Chao1 and Shannon indices were subsequently calculated post-rarefaction using QIIME 1.9.0.

**Host phylogeny and microorganism compositional congruency analyses**

Complete mitochondrial sequences of the 14 studied species were downloaded from GenBank and aligned using Clustal-Omega 1.2.1 (Sievers et al., 2011). The most appropriate nucleotide substitution model was chosen using JModelTest 2.1.10 (Posada, 2008). Bayesian phylogenetic trees were generated in two independent MCMC chains using Beast 2.4 (Drummond, Suchard, Xie, & Rambaut, 2012), with an uncorrelated log-normal relaxed molecular clock, a Yule speciation prior and 5 million generations sampled every 1000 states.  MCMC chains were visualized in Tracer 1.5 to ensure that both independent chains had converged and the estimated sample size (ESS) values of all parameters were above 500. TreeAnnotator 2.4 was used to generate a maximum clade credibility tree using median node heights and 20% burn-in.

Microbiota composition dendrograms were generated using UniFrac distances by a custom pipeline that incorporated the phylogenetic uncertainty of microorganisms. Bayesian phylogenetic trees of methanogens and acetogens were generated using the same pipeline employed for host mitochondrial genomes. However, instead of generating a maximum clade credibility tree, we randomly sampled 1000 trees from the MCMC chain skipping the 20% of trees as burn-in, and generated UniFrac distance matrices using each of those as phylogenetic reference trees. Distance matrices were transformed into dendrograms using UPGMA hierarchical clustering, and a consensus dendrogram containing node uncertainties was generated using TreeAnnotator 2.4 for visualization.

In order to measure the extent to which microbial composition reflected host phylogenies, we computed a Phylosymbiosis Index (PI), by averaging the topology comparisons of the host phylogenetic tree with the 1000 methanogens/acetogens composition dendrograms generated in the previous step. The index was computed using each of the 1000 phylogenetic trees as the complement of the division between the Robinson-Foulds (RF) topological distance between the trees (RF_i_) and the maximum possible RF distance (RF_max_) for a tree with 14 taxa (1 - (RF_i_/RFmax)). Thus, PI ranges from 0 to 1, where 0 indicates there is no phylogenetic signal in the microbial composition (completely different topology) and 1 indicates perfect phylosymbiosis (identical topology). For visualization, host phylogeny and microbial composition tree topologies were compared using the *tanglegram* function in the R package *dendextend* (Galili, 2015).

***Interpretive analysis between rumen structure and microorganism composition***

We compared the methanogen and acetogen communities with morphological characteristics of the studied species’ rumen. The employed morphological feature was the surface enlargement factor (SEF), which reflects the degree of basal mucosal surface gain due to papillation of the rumen wall (Schnorr & Vollmerhaus, 1967). SEF data of the atrium, dorsal sac and ventral sac of the rumen was obtained from (Clauss, Hofmann, Fickel, Streich, & Hummel, 2009) for all analysed species except *Cervus eldii*, *Cervus albirostris* and *Moschus berezovskii*, which were excluded from the analyses due to lack of information. We compared the species distance matrices generated from microbial composition and SEF data through mantel and Pearson's product moment correlation tests using the R functions *mantel* and *cor.test*. We further analysed the correlation between the relative abundances of the dominant methanogens and the mean SEF values of the dorsal and ventral sacs using *cor.test*.

**Microbial community and metabolite relationship**

To compare the methanogen and acetogen communities in the rumen, across all hosts, Bray-Curtis distance matrices generated to perform principal coordinates analysis (PCoA). Moreover, hierarchical clustering of the methanogens and acetogens based on Pearson’s correlation using Bray-Curtis matrices, were also used to compare the similarities among these communities. Redundancy analysis (RDA) was applied to identify the association of rumen methanogens, acetogens, and metabolites. Significance of variables relating to the communities was evaluated by permutation test (n=999). All statistical analyses were performed using the R package *vegan* (https://CRAN.R-project.org/package=vegan).

Co-occurrence network analysis was used to examine the existence of correlations among the methanogens, acetogens and metabolites across all hosts following our previously published methods (Z. Li et al., 2015). In general, Spearman’s rank correlations and *p*-values were calculated and plotted using the *hmisc* and *corrplot* packages. Statistical *p*-values were corrected using the Benjamini-Hochberg method. A spearman’s correlation greater than 0.5 with a corrected significance level less than 0.05 were selected to construct the network. Network analyses were carried out with Cytoscape 2.8.2 using a force-directed algorithm (Smoot, Ono, Ruscheinski, Wang, & Ideker, 2011). Moreover, the interaction networks were analyzed and compared by DyNet (Goenawan, Bryan, & Lynn, 2016), to uncover how the networks were physically rewired.

All multivariate and community analyses were performed using the *reshape*, *ggplot2*, *coin*, *exactRankTests* and *stats* packages implemented in R. Kruskal–Wallis analysis, was used to test the statistical significance of alpha-diversity indices and the relative abundance of each taxon in all hosts. Significance (*p* < 0.05) was based on the Benjamini-Hochberg corrected *p*-value from the Kruskal-Wallis test. All values were expressed as the mean unless otherwise stated.

**References**

Caporaso, J. G., Kuczynski, J., Stombaugh, J., Bittinger, K., Bushman, F. D., Costello, E. K., Knight, R. (2010). QIIME allows analysis of high-throughput community sequencing data. *Nature Methods, 7*(5), 335-336. doi:10.1038/Nmeth.F.303

Clauss, M., Hofmann, R. R., Fickel, J., Streich, W. J., & Hummel, J. (2009). The intraruminal papillation gradient in wild ruminants of different feeding types: Implications for rumen physiology. *Journal of Morphology, 270*(8), 929-942. doi:10.1002/jmor.10729

Drummond, A. J., Suchard, M. A., Xie, D., & Rambaut, A. (2012). Bayesian phylogenetics with BEAUti and the BEAST 1.7. *Molecular Biology and Evolution, 29*(8), 1969-1973. doi:10.1093/molbev/mss075

Gagen, E. J., Denman, S. E., Padmanabha, J., Zadbuke, S., Al Jassim, R., Morrison, M., & McSweeney, C. S. (2010). Functional gene analysis suggests different acetogen populations in the bovine rumen and tammar wallaby forestomach. *Applied and Environmental Microbiology, 76*(23), 7785-7795. doi:10.1128/aem.01679-10

Galili, T. (2015). dendextend: an R package for visualizing, adjusting and comparing trees of hierarchical clustering. *Bioinformatics, 31*(22), 3718-3720. doi:10.1093/bioinformatics/btv428

Gasteiger, E., Gattiker, A., Hoogland, C., Ivanyi, I., Appel, R. D., & Bairoch, A. (2003). ExPASy: the proteomics server for in-depth protein knowledge and analysis. *Nucleic Acids Research, 31*(13), 3784-3788. doi:10.1093/nar/gkg563

Goenawan, I. H., Bryan, K., & Lynn, D. J. (2016). DyNet: visualization and analysis of dynamic molecular interaction networks. *Bioinformatics, 32*(17), 2713-2715. doi:10.1093/bioinformatics/btw187

Haas, B. J., Gevers, D., Earl, A. M., Feldgarden, M., Ward, D. V., Giannoukos, G., . . . Consortium, H. M. (2011). Chimeric 16S rRNA sequence formation and detection in Sanger and 454-pyrosequenced PCR amplicons. *Genome Research, 21*(3), 494-504. doi:10.1101/gr.112730.110

Li, W., & Godzik, A. (2006). Cd-hit: a fast program for clustering and comparing large sets of protein or nucleotide sequences. *Bioinformatics, 22*(13), 1658-1659. doi:10.1093/bioinformatics/btl158

Li, Z., Wright, A. D. G., Liu, H., Fan, Z., Yang, F., Zhang, Z., & Li, G. (2015). Response of the rumen microbiota of sika deer (*Cervus nippon*) fed different concentrations of tannin rich plants. *PLoS ONE, 10*(5), e0123481. doi:10.1371/journal.pone.0123481

Posada, D. (2008). jModelTest: phylogenetic model averaging. *Molecular Biology and Evolution, 25*(7), 1253-1256. doi:10.1093/molbev/msn083

Schnorr, B., & Vollmerhaus, B. (1967). Das Oberflächenrelief der Pansenschleimhaut bei Rind und Ziege. *Zentralblatt für Veterinärmedizin Reihe A, 14*(2), 93-104. doi:10.1111/j.1439-0442.1967.tb00219.x

Seedorf, H., Kittelmann, S., Henderson, G., & Janssen, P. H. (2014). RIM-DB: a taxonomic framework for community structure analysis of methanogenic archaea from the rumen and other intestinal environments. *PeerJ, 2*, e494. doi:10.7717/peerj.494

Sievers, F., Wilm, A., Dineen, D., Gibson, T. J., Karplus, K., Li, W., . . . Higgins, D. G. (2011). Fast, scalable generation of high-quality protein multiple sequence alignments using Clustal Omega. *Molecular Systems Biology, 7*, 539-539. doi:10.1038/msb.2011.75

Smoot, M. E., Ono, K., Ruscheinski, J., Wang, P. L., & Ideker, T. (2011). Cytoscape 2.8: new features for data integration and network visualization. *Bioinformatics, 27*(3), 431-432. doi:10.1093/bioinformatics/btq675

# Supplementary Figures and Tables

##
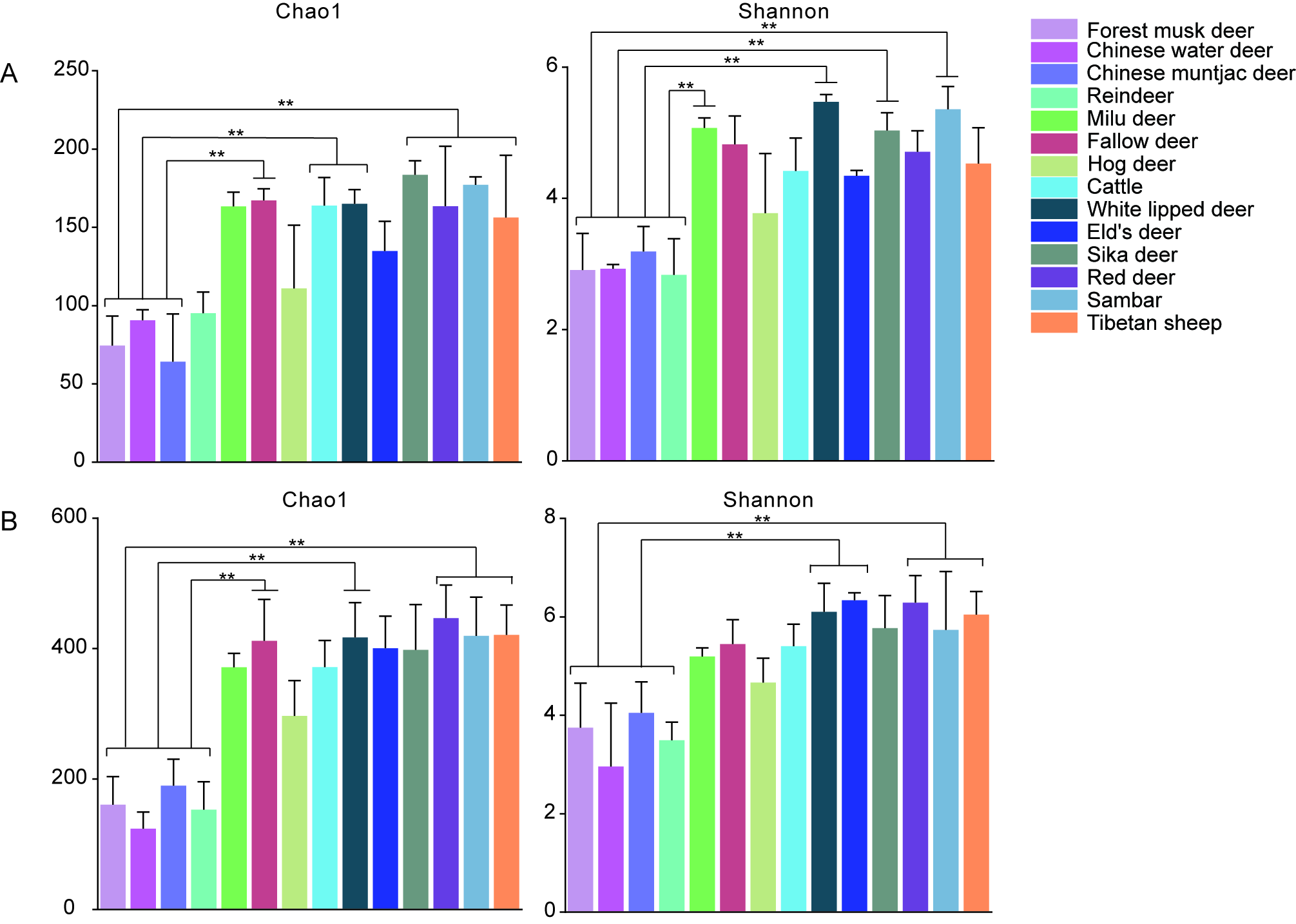
Supplementary Figures

**Supplementary Figure 1.** . The diversity and richness indices of acetogens (A) and methanogens (B) in the rumen of 14 species.* *p*<0.05, ** *p*<0.01, *** *p*<0.001.


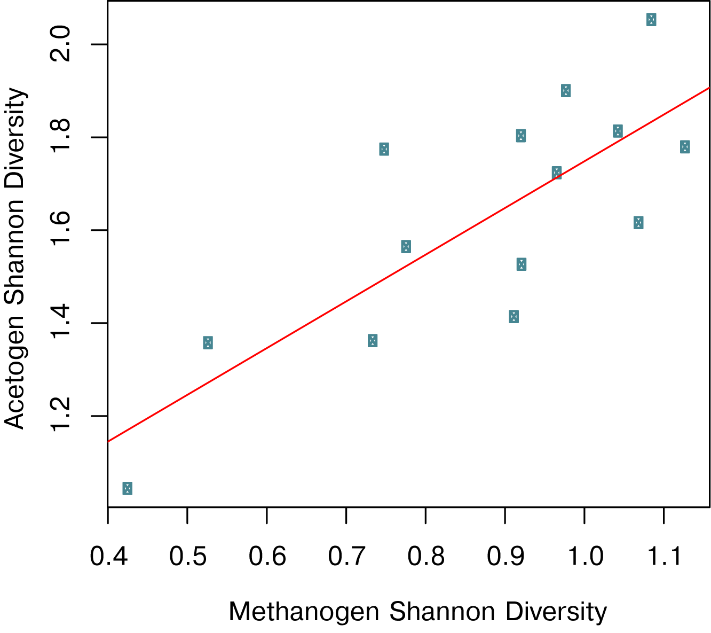
**Supplementary Figure 2.** Correlation of the Shannon diversity index of methanogen and acetogen communities in the rumen of 14 species.


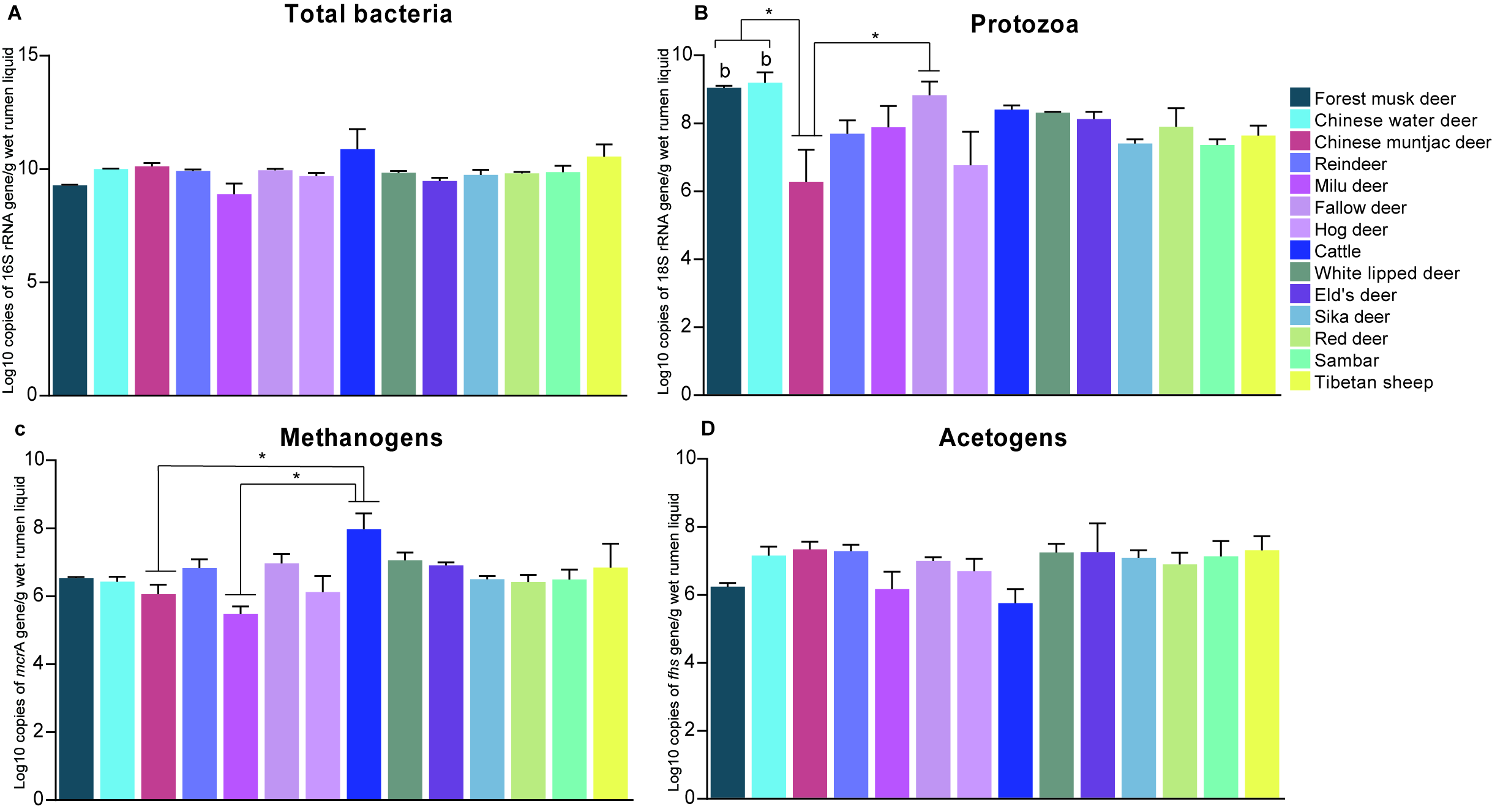


**Supplementary Figure 3.** Density of the selected microbes in rumen of 14 ruminant species including total bacteria (A), protozoa (B), methanogens (C) and acetogens (D) Abundance was expressed as log10 copies of target gene per gram of wet weight. Target gene: 16S rRNA gene for bacteria; mcrA for methanogens; 18S rRNA for protozoa; fhs for acetogens. The different letters (a, b) denote significant differences (Kruskal-Wallis tests, FDR-adjusted q<0.05).
