# Supplementary material for: Comparative Microbiome Analysis Reveals the Ecological Relationships Between Rumen Methanogens, Acetogens, and Their Hosts
Source: Front Microbiol. 2020 Jun 30;11:1311. doi: 10.3389/fmicb.2020.01311 (PMC7344211; doi:10.3389/fmicb.2020.01311)
Supplement: TABLE S1 — The distribution, habit, diet, and management of the ruminant species used in the present study. [file Table_1.DOCX]

**Table S1. The distribution, habit, diet and mangement of the ruminant species used in the present study.**

| **Species** | **Common name** | **Number of animals** | **Source** | **Mean BW** | **Diet** | **Management** |
| --- | --- | --- | --- | --- | --- | --- |
| *Cervus albirostris* | White lipped deer | 8 | Qilian Mountain, Xining, Qinghai, China | 192 kg | Grasses, herbs, lichens, leaves and bark of trees, and bushes. | The animals were maintained by the Tibetan people, with frees access to grass distributed in the Qilian mountain. |
| *Cervus nippon* | Sika deer | 5 | Qilian deer farm, Xining, Qinghai, China | 120 kg | Grasses, herbs, leaves and bark of trees, and bushes. | The animals were reared by the farm freely grazing the pasture on the Qilian mountain. |
| *Cervus elaphus* | Red deer | 5 | Qilian deer farm, Xining, Qinghai, China | 223 kg | Sedges, Kobresia myosuroides, and Polygonum viviparum. | The animals were reared by the farm freely grazing the pasture on the Qilian mountain. |
| *Dama dama* | Fallow deer | 8 | Beijing Milu ecological research center, Beijing, China | 72 kg | Corn silage, alfalfa and concentrate diets. | Thirty animals were placed in a individual pen with free access to water, and were supplied the diets. |
| *Hydropotes inermis* | Chinese water deer | 8 | Local farm at Yancheng city, Jiangsu, China | 15 kg | Ryegrass and sweet potato leaf. | Twenty animals were placed together in an individual pen. |
| *Muntiacus reevesi* | Chinese muntjac deer | 8 | Local farm at Ji'an city, Jiangxi, China | 17 kg | Seeds, bark, fruits, and foliage. | The animals were free to digest the plants and fruits in the mountain of the farm. |
| *Elaphurus davidianus* | Pere David’s deer | 6 | Beijing Milu ecological research center, Beijing, China | 289 kg | Grass including yard grass, Speedwell, and Crabgrass. | The animals were free to digest the plants in the zoo and water. |
| *Cervus eldii* | Eld's deer | 5 | Datian Nature Reserve of Hainan Eld's deer, Haikou, Hainan, China | 119 kg | Herbaceous plant, such as Lygodium japonicum, Polyalthia suberosa, Imperata cylindrica. | The animals were free to digest the plants in the reserve zone. |
| *Rusa unicolor* | Sambar | 8 | Fengmu deer farm, Haikou, Hainan, China | 252 kg | Corn silage, alfalfa, and concentrate diets | Thirty animals were maintained in a individual pen with free access to water. The animals were fed twice each day. |
| *Axis porcinus* | Hog deer | 6 | Chengdu Zoological Garden, Chengdu, Sichuan, China | 37 kg | Rye grass, oat grass and alfalfa. | Twenty animals were maintained in Chengdu Zoological Garden with free access to water. The animals were fed twice each day. |
| *Rangifer tarandus* | Reindeer | 8 | Greater Khingan Mountains, Inner Mon-  golia Autonomous Region, China | 130 kg | Lichen. | The domesticated herd maintained by Ewenki hunter-herders. |
| *Moschus berezovskii* | Forest musk deer | 7 | Sichuan Institute of Musk Deer Breeding, Chengdu, Sichuan, China | 9 kg | Lettuce, carrot, and pumpkin | Each animal was maintained at the individual pen. The animals were fed twice each day with free access to water. |
| *Ovis aries* | Tibet sheep | 8 | Qilian deer farm, Xining, Qinghai, China | 26 kg | Tibet pasture. | Free range. |
| *Bos taurus* | Cattle | 8 | Animal farm of Nanjing agriculture university, Nanjing city, Jiangsu, China | 607  kg | Alfalfa and concentrate diets | Each animal was maintained in an individual pen with free access to water. All animals were fed twice each day. |
