# Supplementary material for: Comparative Microbiome Analysis Reveals the Ecological Relationships Between Rumen Methanogens, Acetogens, and Their Hosts
Source: Front Microbiol. 2020 Jun 30;11:1311. doi: 10.3389/fmicb.2020.01311 (PMC7344211; doi:10.3389/fmicb.2020.01311)
Supplement: TABLE S2 — Primer sets used to quantify the rumen microbiota of 14 ruminant species. [file Table_2.DOCX]

**Table S2. Primer sets used to quantify the rumen microbiota of 14 ruminant species**

| **Target species/genes** | **Primer sequences** | **Product size**  **(bp)** | **PCR efficiency (%)** | **References** |
| --- | --- | --- | --- | --- |
| Total bacteria | Forward: 5^'^-CGGCAACGAGCGCAACCC-3^'^ | 143 | 104 | Denman and McSweeney, 2006 |
|  | Reverse: 5^'^-CCATTGTAGCACGTGTGTAGCC-3^'^ |  |  |  |
| Protozoa | Forward: 5^'^-GCTTTCGWTGGTAGTGTATT-3^'^ | 234 | 108 | Sylvester et al., 2004 |
|  | Reverse: 5^'^-CTTGCCCTCYAATCGTWCT-3^'^ |  |  |  |
| *mcr*A | Forward: 5^'^-TTCGGTGGATCDCARAGRGC-3^'^ | 160 | 104 | Denman et al., 2007 |
|  | Reverse: 5^'^-GBARGTCGWAWCCGTAGAATCC-3^'^ |  |  |  |
| *fhs* | Forward: 5^'^-GTWTGGGCWAARGGYGGMGAAGG-3^'^ | 342 | 104 | Xu et al., 2009 |
|  | Reverse: 5^'^-GARGAYGGWTTTGAYATYAC-3^'^ |  |  |  |

**Reference**

Denman, S.E., Tomkins, N.W., and McSweeney, C.S. (2007). Quantitation and diversity analysis of ruminal methanogenic populations in response to the antimethanogenic compound bromochloromethane. FEMS Microbiol.Ecol.62, 313-322. j.1574-6941.2007.00394.x

Denman, S.E., and McSweeney, C.S. (2006). Development of a real‐time PCR assay for monitoring anaerobic fungal and cellulolytic bacterial populations within the rumen. FEMS Microbiol. Ecol. 58, 572-582.

Sylvester, J.T., Karnati, S.K., Yu, Z. T., Morrison, M., and Firkins, J.L. (2004). Development of an assay to quantify rumen ciliate protozoal biomass in cows using real-time PCR. J. Nutr. 134,3378-3384.

Xu, K., Liu, H., Du, G., and Chen, J. (2009). Real-time PCR assays targeting formyltetrahydrofolate synthetase gene to enumerate acetogens in natural and engineered environments. Anaerobe 15, 204-213.doi: 10.1016/j.anaerobe.2009.03.005
