# Supplementary material for: Comparative Microbiome Analysis Reveals the Ecological Relationships Between Rumen Methanogens, Acetogens, and Their Hosts
Source: Front Microbiol. 2020 Jun 30;11:1311. doi: 10.3389/fmicb.2020.01311 (PMC7344211; doi:10.3389/fmicb.2020.01311)
Supplement: TABLE S3 — Comparison of the percentage of rumen volatile fatty acids (VFAs) among the 14 ruminant species. [file Table_3.DOCX]

**Table S3**. The molar percentage of VFA in the rumen among the 14 ruminant species.

| Item | Acetate | Propionate | Butyrate | Valerate | Isobutyrate | Isovalerate | Total VFA (mM) |
| --- | --- | --- | --- | --- | --- | --- | --- |
| White lipped deer | 69.54±0.42 | 13.01±0.29 | 12.44±0.25 | 1.18±0.05 | 1.4±0.06 | 2.43±0.16 | 81.37±14.25 |
| Cattle | 66.06±2.39 | 15.29±0.36 | 9.89±0.53 | 2.11±0.9 | 3.68±1.4 | 2.98±1.45 | 93.33±18.90 |
| Red deer | 74.71±1.63 | 13.86±0.33 | 8.78±1.27 | 0.62±0.1 | 0.87±0.06 | 1.16±0.12 | 76.51±7.33 |
| Chinese water deer | 65.28±1.32 | 10.93±1.44 | 17.09±1.11 | 1.05±0.12 | 2.35±0.44 | 3.3±0.48 | 65.76±14.50 |
| Chinese muntjac deer | 57.27±5.45 | 16.20±2.61 | 8.61±1.28^bc^ | 4.69±1.34 | 6.34±2.39 | 6.89±2.56 | 62.36±11.45 |
| Sika deer | 70.18±0.74 | 14.53±0.28 | 10.35±0.62 | 1.21±0.16 | 1.44±0.24 | 2.29±0.39 | 83.31±12.66 |
| Forest musk deer | 71.53±2.99 | 9.09±2.38 | 10.86±1.45 | 2.01±0.18 | 2.89±0.49 | 3.63±0.61 | 62.00±5.69 |
| Milu deer | 77.18±2.22 | 10.72±1.84 | 6.73±0.59 | 0.78±0.13 | 1.65±0.13 | 2.94±0.35 | 72.07±23.77 |
| Eld's deer | 83.80±0.08 | 10.96±0.09 | 4.77±0.04 | 0.09±0.02 | 0.12±0.05 | 0.26±0.06 | 86.89±20.09 |
| Sambar | 72.67±0.38 | 17.05±0.35 | 8.91±0.21 | 0.54±0.04 | 0.37±0.02 | 0.47±0.04 | 69.45±14.25 |
| Fallow deer | 71.04±1.83 | 14.17±0.97 | 9.71±0.96 | 1.52±0.14 | 1.29±0.06 | 2.27±0.12 | 58.56±6.41 |
| Hog deer | 71.06±1.12 | 14.31±1.50 | 8.8±0.38 | 0.99±0.12 | 1.69±0.21 | 3.14±0.36 | 84.26±6.69 |
| Reindeer | 69.01±1.49 | 17.63±1.03 | 6.74±1.06 | 1.04±0.11 | 2.18±0.19 | 3.39±0.25 | 70.77±10.42 |
| Tibetan sheep | 68.82±0.19 | 14.36±0.56 | 13.24±0.53 | 0.95±0.06 | 1.11±0.05 | 1.52±0.08 | 78.33±13.16 |

Note. The data is presented as Mean ± SEM. The detailed comparison result is listed in Table S4.
